# A 23-year bibliometric analysis of the development of global research on hereditary renal carcinoma

**DOI:** 10.3389/fonc.2024.1364997

**Published:** 2024-06-03

**Authors:** Xiaopeng Lan, Mei Feng, Ji Lv, Luchen Zhang, Pengcheng Hu, Yizhen Wang, Yanhui Zhang, Shen Wang, Chunzhao Liu, Chunlei Liu

**Affiliations:** ^1^ Department of Urology, Qingdao Central Hospital, University of Health and Rehabilitation Sciences, Qingdao, China; ^2^ Institute of Biochemical Engineering, College of Materials Science and Engineering Qingdao University, Qingdao, China

**Keywords:** bibliometric analysis, hereditary renal cancer, Hippel-Lindau disease, hereditary leiomyomatosis and renal cell carcinoma, hereditary papillary renal cell carcinoma, tuberous sclerosis complex, Birt Hogg Dube syndrome

## Abstract

**Objectives:**

Medical research continues to be extensively devoted to investigating the pathogenesis and treatment approaches of hereditary renal cancer. By aspect including researchers, institutions, countries, journals, and keywords, we conduct a bibliometric analysis of the literature pertaining to hereditary renal cancer over the last 23 years.

**Methods:**

From the Web of Science Core Collection, we conducted a search for publications published between January 1, 2000 and November 28, 2023. Reviews and original articles were included.

**Results:**

A cumulative count of 2,194 publications met the specified criteria for inclusion. The studies of the included articles involved a collective of 2,402 institutions representing 80 countries. Notably, the United States exhibited the highest number of published documents, constituting approximately 45.49% of the total. The preeminent institution in this discipline is the National Cancer Institute (NCI), which maintains a publication volume of 8.98%. In addition to being the most prolific author (125 publications), Linehan WM’s works received the highest number of citations (11,985). In a comprehensive count, 803 journals have published related articles. In the top 10 most recent occurrences were the terms “hereditary leiomyomatosis” and “fumarate hydratase.”

**Conclusion:**

This is the first bibliometric analysis of the literature on hereditary renal cancer. This article offers a thorough examination of the present status of investigations concerning hereditary renal cancer during the previous 23 years.

## 1 Introduction

With an estimated 81,800 new cases diagnosed with renal cell carcinoma (RCC) in the United States in 2023, it is the eighth most prevalent kind of cancer overall (1). Although there has been a consistent yearly rise in the incidence of RCC and a recognized shift in the stage at which it is diagnosed (2), the literature still indicates that more than 20% of cases are first diagnosed as metastatic RCC (3). An estimated 5–8% of renal malignancies are hereditary renal cancers (HRNs), although this may be an underestimate due to uncharacterized genetic factors (4). These tumors carry a unique spectrum of pathologic and molecular alterations, the knowledge of which has been expanding in recent years. Indebted to this knowledge, many advances in the treatment of these tumors have been achieved (5). There are numerous etiologies for hereditary kidney cancer, including von Hippel-Lindau (VHL) disease, hereditary leiomyomatosis and renal cell carcinoma (HLRCC), hereditary papillary renal cell carcinoma type 1 (HPRCC1), Tuberous Sclerosis Complex 1/2 (TSC1/2), Birt-Hogg-Dube disease (BHD), and other related genetic disorders (6).

The most common symptoms of VHL syndrome are hemangioblastoma in the brain and spinal cord, hemangioblastoma in the retina, renal carcinoma or cyst, pancreatic tumor or cyst, adrenal pheochromocytoma, endolymphatic cyst, reproductive system cyst, and others. The syndrome happens about once every 36,000 people. RCC is the most significant clinical sign and leading cause of mortality for individuals with VHL syndrome (7). Bilateral and multifocal renal carcinomas occur in almost half of VHL patients. 25% to 60% of patients with VHL syndrome develop RCC, with an average age of onset between 40 and 45 years; this is approximately 20 years earlier than the average age of onset for sporadic renal cancer (8). With advancing age, the patient will experience a constant occurrence of new RCC in both kidneys, resulting in the presence of multiple tumors. The majority of RCC seen in individuals with VHL syndrome is of the clear cell carcinoma subtype, with a predominance of grade I histological grade (9). The renal cysts linked to VHL syndrome primarily manifest as bilateral numerous renal cysts, which may be categorized as simple renal cysts, atypical proliferative renal cysts, or cystic clear cell carcinoma. Renal cysts in VHL syndrome have been shown by some investigations to be precancerous lesions of renal cell carcinoma (10).

Hereditary leiomyomatosis and renal cell carcinoma (HLRCC) are predominantly prevalent among young women, with the majority of renal tumors being detected at an early stage, appearing on one side only, and consisting of a single focal point. From a clinical perspective, these tumors exhibit a strong inclination to rapidly spread to distant locations, especially in cases of tiny tumors, resulting in a bleak prognosis. Simultaneously, these patients have a tendency to develop cutaneous leiomyoma as well as numerous and early-onset uterine myomas (11). Cutaneous leiomyomas typically manifest as numerous firm nodules on the torso and limbs, often exceeding 10 or even 100 in number, with a size ranging from 0.4 to 2.5 mm. These nodules may exhibit a segmental pattern and are commonly associated with symptoms such as pain and paraesthesia. The typical age at which skin lesions first appear is approximately 25 years. Uterine leiomyomas, also known as uterine fibroids, are more common in people with cutaneous leiomyomas. Patients may present with a multitude of uterine fibroids, with a maximum count of 20, varying in size from 1.5 to 10 cm (12). In contrast to other hereditary renal carcinomas, hereditary papillary renal carcinoma (HPRC) exclusively affects the kidney and does not involve any other organs. HPRC frequently manifests as bilateral, numerous tumors, with some cases even reporting the presence of hundreds of tiny lesions. Patients with advanced stages of chronic renal failure frequently experience a variety of clinical symptoms associated with uremia. Because there are no other symptoms outside of the kidneys, the clinical symptoms of these patients resemble those of typical renal cancer. It is important to examine the likelihood of hereditary kidney cancer in individuals under the age of 30 who have a family history of HPRC type I (13, 14).

Unlike other hereditary tumor disorders, hereditary renal carcinoma syndrome displays a diverse array of clinical and molecular features, impacting numerous organs. This necessitates the involvement of not only clinical disciplines but also other fundamental disciplines such as molecular diagnosis, genetics, and bioinformatics in the diagnosis and treatment process. This interdisciplinary approach is essential for the development of comprehensive diagnosis, monitoring, and treatment protocols. Thus, for cases of hereditary renal cancer, the preferred approach is to conduct diagnostics and therapy using a multidisciplinary approach (15, 16). The clinical treatment benefits of patients can be enhanced by fully utilizing the advantages of several disciplines based on the patient’s condition. The field of hereditary renal carcinoma has shown significant growth in research activity, with a substantial number of articles being published annually. Researchers must acquire proficiency in understanding research trends and diligently track the most significant recent advancements, despite the difficulties involved. Hence, it is necessary to conduct a thorough and measurable examination that highlights current areas of interest and proposes future research avenues.

To promptly ascertain the advancements and future prospects of a particular scientific domain, an examination of the published literature is essential. The advent of the Internet and novel publishing techniques have facilitated the retrieval and investigation of documents. However, this has also introduced the challenge of managing vast resources. Bibliometric analysis is an appropriate approach for doing a thorough examination of a complete academic topic, encompassing a large number of publications (17). Through the software, literature metrology conducts clustering and other operations. A number of iterations (analysis, data cleansing, and re-analysis) may be required before the generated clustering can be utilized to evaluate the research topic. Concurrently, documents pertaining to associated subjects have been compiled. By conducting bibliometric analysis iteratively, it is possible to identify fundamental subdivision themes within the field and compile a collection of documents pertaining to each theme; this information is extremely useful for subsequent literature reviews. There is currently no bibliometric analysis of hereditary kidney carcinoma. Hence, it is imperative to conduct a thorough, current, and practical bibliometric analysis of hereditary renal carcinoma.

The present bibliometric analysis identified all the articles published between January 1, 2000, and November 28, 2023, that were directly related to hereditary renal carcinoma. The aim of this study was to provide a comprehensive overview of this field, including significant developments, research trends, and current areas of interest for scholars. Researchers can not only identify significant publications, journals, and potential collaborators on the basis of this study, but they may also be inspired to devise additional investigations.

## 2 Methods

### 2.1 Database and search strategy

Our research utilizes the Science Citation Index Expanded (SCI-Expanded) from the Web of Science Core Collection (WoSCC) of Clarivate Analytics as the data source. Containing articles from close to 9,000 high-impact journals, the WoSCC is an extensively utilized database for bibliometric research.

On November 28, 2023, a search for publications pertaining to hereditary renal carcinoma was conducted. The formulation of the search query was as follows: topic = “hereditary OR inherited OR familial” AND “(renal OR kidney) NEAR/5 (cancer OR tumor OR oncology OR neoplasm OR carcinoma)” AND “publication date = (January 1, 2000–November 28, 2023)”. The data, which included the annual study numbers, countries, institutions, authors, journals, citations, and keywords, was extracted independently by two authors (Xiaopeng Lan and Mei Feng). A discussion with a third reviewer was employed to reconcile the differences that existed between the two reviewers. The downloaded search results were presented in both plain text and BibTex, specifically in the “Full Records and Cited References” section. Moreover, the scope of publications was restricted to scholarly reviews and original articles, and the language utilized was exclusively English.

### 2.2 Data analysis and visualization

In order to guarantee the precision of the data and the dependability of the research, two researchers autonomously retrieved and examined the data. Co-authorship, co-occurrence, and co-citation analysis are the primary and most important markers in bibliometric analysis. Co-authorship analysis is performed to examine the connections between authors, countries, or institutions. Co-occurrence analysis is a quantitative technique used to examine the things that appear most frequently in articles. A co-citation analysis was performed by comparing the ranked outcomes with the co-citation score. The data gathered from articles was analyzed and summarized separately by two authors, and then reviewed and validated by a third author. In the event that the writers disagree, the authors were decided via negotiation. Categories of literature, including meeting abstracts, editorial materials, proceeding papers, book chapters, and correspondence, were omitted from the documents obtained through the search process. Retained were exclusively original articles and reviews. Meanwhile, to ensure that the data analysis procedure did not contain any inaccurate information, only English-language articles were incorporated.

Data visualization for bibliometric analysis was conducted utilizing R language software (4.2.3), CiteSpace (version 6.2.R4), and VOSviewer (version 1.6.20). The variables in this descriptive study were represented by numerical values and percentages. Due to the absence of comparisons, P-values were not computed.

The R package bibliometrix was utilized to extract data on keywords, countries, and years. Subsequently, heat maps were generated to visualize the frequency of nation-wide publications over time. Furthermore, this study employs VOSviewer, a bibliometric tool that utilizes distance-based techniques to visualize bibliometric networks. Specifically, VOSviewer is utilized to analyze and visualize nation, institution, journal, and author collaboration networks, as well as perform keyword overlay analysis. The settings for VOSviewer were configured as follows: the counting method used is the full counting approach. However, documents with a large number of nations, institutions, or authors are ignored. The maximum number allowed for each category per document is 25. Keyword co-occurrence, clustering, and burst were all visualized using CiteSpace because of its ability to dynamically display changing bibliometric networks. Here are the CiteSpace parameters: link retention factor = 2, look back years = -1, e for top *N* = 2, time period = 2000–2023, years per slice = 1, selection criteria = top 50.

Patient consent and clinical investigations were excluded from this bibliometric investigation. As a result, the institutional review board and ethics committee were unnecessary.

### 2.3 Patient and public involvement

Patients and the public were not involved in any way in this study.

## 3 Results

### 3.1 The trend of annual publication outputs

**Figure 1:**
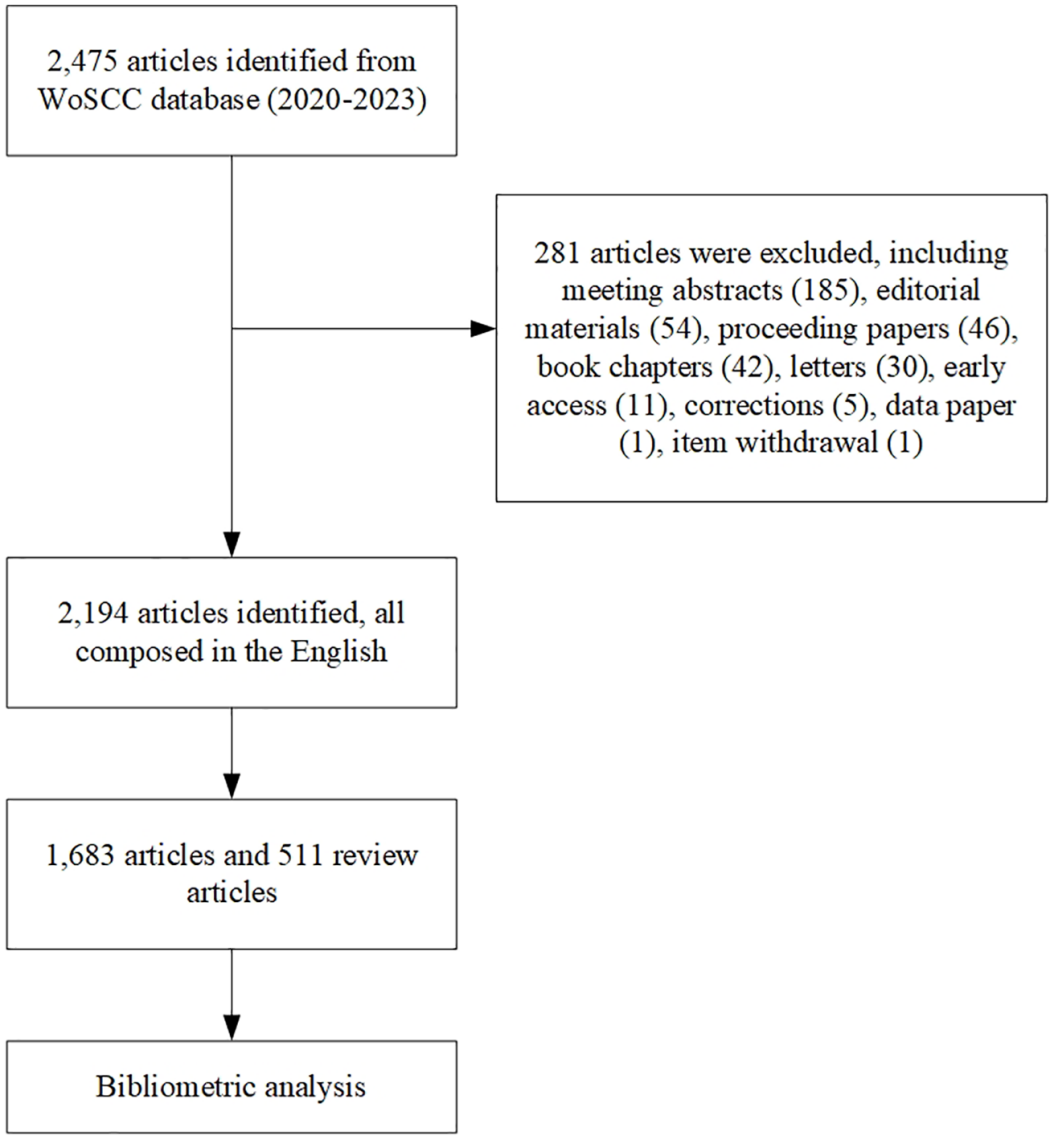
Flow chart of the present study.

**Figure 2:**
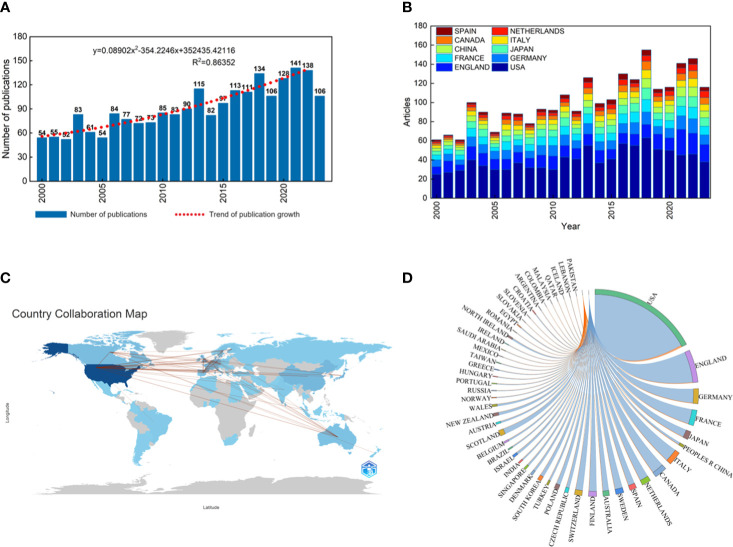
**(A)** Global trends in the publication of hereditary renal carcinoma. The dotted line shows the annual publication growth trend. Equation: y = 0.08902x2–354.2246x+352435.42116, R2 = 0.86352, can predict the annual documents. **(B)** Publications’ dynamic characteristics in the top ten nations from 2000 to 2023. **(C)** A world map visualization of the relationships between publications and collaborations. **(D)** Network mapping of international collaboration based on 2194 papers.

As a consequence of the infrequent occurrence of hereditary renal carcinoma, the corpus of published literature is not notably extensive. As illustrated in our flowchart, the retrieval method yields a preliminary count of 2,475 documents retrieved. Including meeting abstracts, editorial materials, proceeding papers, book chapters, letters, and other categories of literature. The remaining 2,194 articles were exclusively composed in the English language. 1,683 articles and 511 review articles were included (**Figure 1**). The annual publication volume is a quantitative measure that indicates the level of interest in a certain academic subject. As illustrated by the bar chart in **Figure 2A**, a total of 2,194 publications, spanning from 2000 to 2023, satisfied the criterion for inclusion. During the last 20 years, the quantity of publications has fluctuated, although it has displayed a general rising trajectory, suggesting a growing interest among experts in this topic. In addition, the generalized additive model, shown by the red dotted line in **Figure 2A**, was employed to assess the correlation between the quantity of papers and the year of publication. The results indicated that the model exhibited a relatively high level of conformity with the yearly distribution pattern of the literature (*R* ^2 = ^0.863). Additionally, the forecast curve eloquently illustrates the annual increase in publication volume and the upward trend that is likely to persist in the coming years.

### 3.2 Analysis of productive countries/regions

A total of 2,402 organizations from 80 countries participated in the studies of the included articles, with the United States obviously having the most published documents, accounting for approximately 45.49% (**Table 1**). Other countries with a large number of articles include England, Germany, Japan, and France. The United States also has the highest cumulative number of citations, up to 65,823, far exceeding other countries. Literature in English, French, German, and Italian is also most commonly quoted. England is at the top in terms of the number of citations per paper, followed by the United States, which has the most publications. Canada and Italy have the most citations for a single article, despite the fact that their overall number of publications does not rank in the top five. When we break down the publications from the top ten nations, the United States remains the country with the most articles each year (**Figure 2B**).

**Table 1:** Top 10 productive countries/regions associated with hereditary renal carcinoma.

Rank	Country/Region	Publications	Percentage	Total citations	Average article citation
1	USA	998	45.49%	65823	65.95
2	England	235	10.71%	19537	83.14
3	Germany	203	9.25%	8871	43.70
4	Japan	180	8.20%	4882	27.12
5	France	170	7.75%	9280	54.59
6	People R China	149	6.79%	1482	9.95
7	Italy	143	6.52%	8305	58.08
8	Canada	123	5.61%	7556	61.43
9	Netherlands	112	5.10%	5402	48.23
10	Spain	86	3.92%	4085	47.50

### 3.3 Collaboration of countries

**Figure 3:**
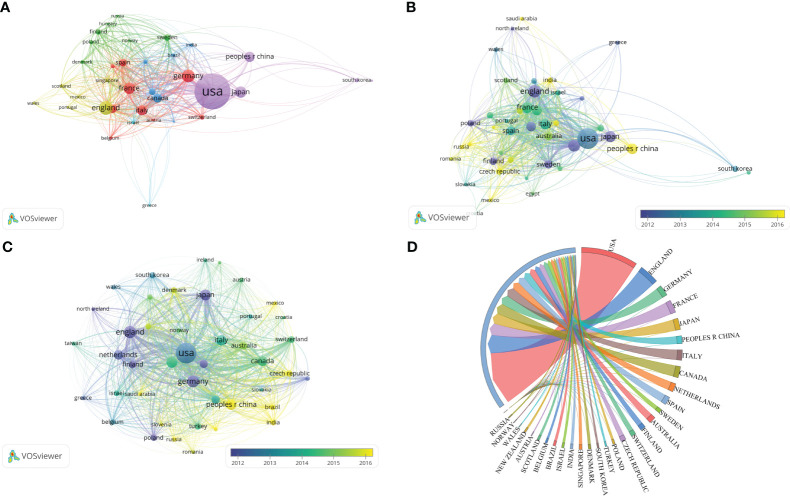
**(A)** Correlations among the countries/regions with more than 10 articles. VOSviewer was responsible for creating the graph. The line thickness indicates the citation strength. **(B)** Network visualization map of collaboration relationships and average publication year of the various countries and regions. **(C)** Network visualization map of cooperation connection and average publication year of the nations and regions of the top 100 publications. **(D)** Network mapping of cooperation among the top 100 articles.

The majority of research was undertaken by writers from a single country. The collaboration between countries and regions was depicted using a collaborative network globe map (**Figure 2C**). North American and European countries collaborated more frequently than others. As shown in a chordal graph (**Figure 2D**), the United States partnered with the majority of countries and regions. A VOSviewer-generated image (**Figure 3A**) depicts the national collaboration network among 35 nations with more than 10 papers published. The thickness of the line indicates the strength of international collaboration, referred to as total link strength (TLS. The top five TLSs were the United States, England, Germany, France, and Japan. The collaboration relationships and average publication year of the various nations and regions were displayed on a network visualization map (**Figure 3B**). The cooperation connection and average publication year of the nations and regions of the top 100 publications were shown on a network visualization map (**Figure 3C**). Out of all the publications on hereditary renal carcinoma, international cooperation was more prevalent among the top 100 articles (**Figure 3D**).

### 3.4 Analysis of productive institutions

**Figure 4:**
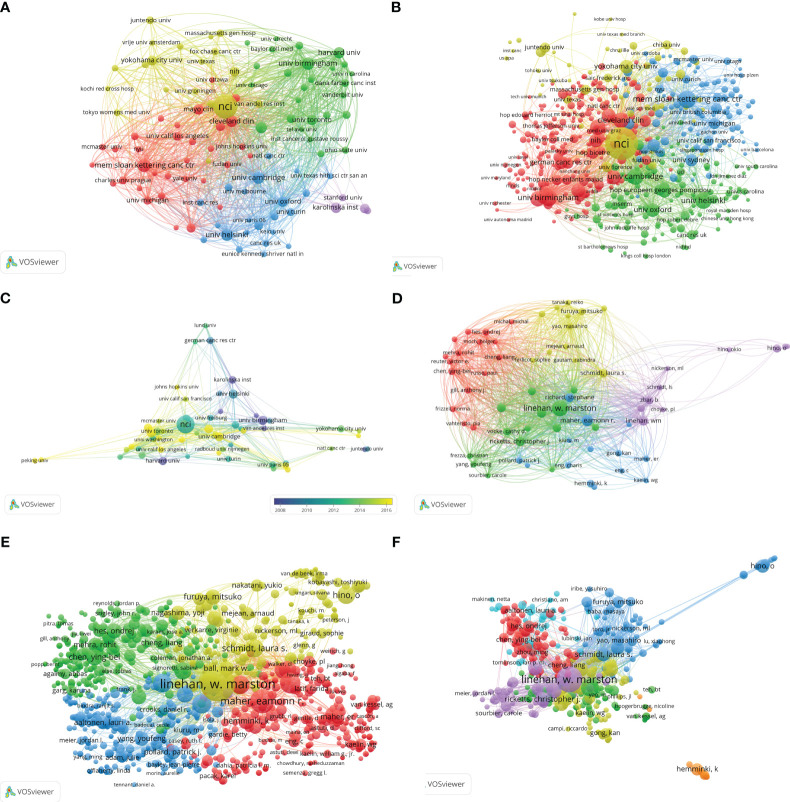
**(A)** Network visualization map of collaboration relationships of institutions with at least 10 papers. **(B)** Network visualization map of collaboration relationships of institutions with at least 3 papers. **(C)** Network visualization map of cooperation connection and average publication year of the institutions with more than 15 publications. **(D)** Visualization map of co-cited authors with more than 10 papers generated by VOSviewer. **(E)** Visualization map of co-cited authors with more than 2 papers generated by VOSviewer. **(F)** Visualization map of co-cited authors generated by VOSviewer.

The United States is home to six of the top ten publications pertaining to hereditary kidney cancer (**Table 2**). With a publication volume of 8.98%, the National Cancer Institute (NCI) is the preeminent institution in this field. Articles from NCI received the most citations. Given that the quality of publications can be more accurately assessed by the number of citations per paper, articles from Harvard University exhibit superior quality, as evidenced by a count of 138.36 citations per paper. Additionally, articles from the University of Birmingham also have a high number of citations per article. On network visualization maps (**Figure 4A, B**), the collaboration relationships of the various institutions with at least 10 papers or at least 3 papers were depicted. The cooperation connection and average publication year of the institutions with more than 15 publications were shown on a network visualization map (**Figure 4C**).

**Table 2:** Top 10 productive institutions associated with hereditary renal carcinoma.

Rank	Institution	Country	Publications	Percentage	Citations	Citations per paper
1	NCI	USA	197	8.98%	16109	81.77
2	Mem Sloan Kettering Canc Ctr	USA	57	2.60%	5439	95.42
3	Univ Birmingham	UK	52	2.37%	5876	113.00
4	Univ Helsinki	Finland	50	2.28%	4169	83.38
5	Univ Cambridge	UK	48	2.19%	3578	74.54
6	Cleveland Clin	USA	44	2.01%	2892	65.73
7	Univ Texas Md Anderson Canc Ctr	USA	43	1.96%	1779	41.37
8	Harvard Univ	USA	42	1.91%	5811	138.36
9	Karolinska Inst	Sweden	37	1.69%	1336	36.11
10	Mayo Clin	USA	36	1.64%	1261	35.03

### 3.5 Analysis of authors

The 2,194 papers on hereditary kidney cancer were authored by 1,000 researchers in total. In this field, Linehan WM was the most productive author (125 papers), with the greatest number of citations (11,985 citations) to his works (**Table 3**). Besides, Maher ER, Srinivasan R, Ricketts CJ, and Merino MJ were also among the top five most prolific writers in the previous 23 years. Notably, Zbar B was the second-cited author, despite having only authored 24 articles in this field. The United States is at the forefront of this area of study, as shown by the seven American writers in the top 10. Collaboration networks and clustering analysis of authors with more than 10 or 2 papers were carried out (**Figures 4D, E**). The collaboration amongst co-cited authors is shown (**Figure 4F**).

**Table 3:** Top 10 productive authors related to hereditary renal carcinoma.

Rank	Author	Country	Publications	Citations	Citations per paper	H-Index
1	Linehan, W. Marston	USA	125	11985	95.88	130
2	Maher, Eamonn R.	UK	45	2862	63.60	22
3	Srinivasan, Ramaprasad	USA	31	2376	76.65	34
4	Ricketts, Christopher J.	USA	29	1618	55.79	6
5	Merino, Maria J.	USA	28	1708	61.00	82
6	Bratslavsky, Gennady	USA	27	1454	53.85	40
7	Richard, Stephane	France	27	1830	67.78	78
8	Schmidt, Laura S.	USA	26	2373	91.27	46
9	Hino, O	Japan	25	717	28.68	46
10	Zbar, B	USA	24	4580	190.83	67

### 3.6 Analysis of journals

**Figure 5:**
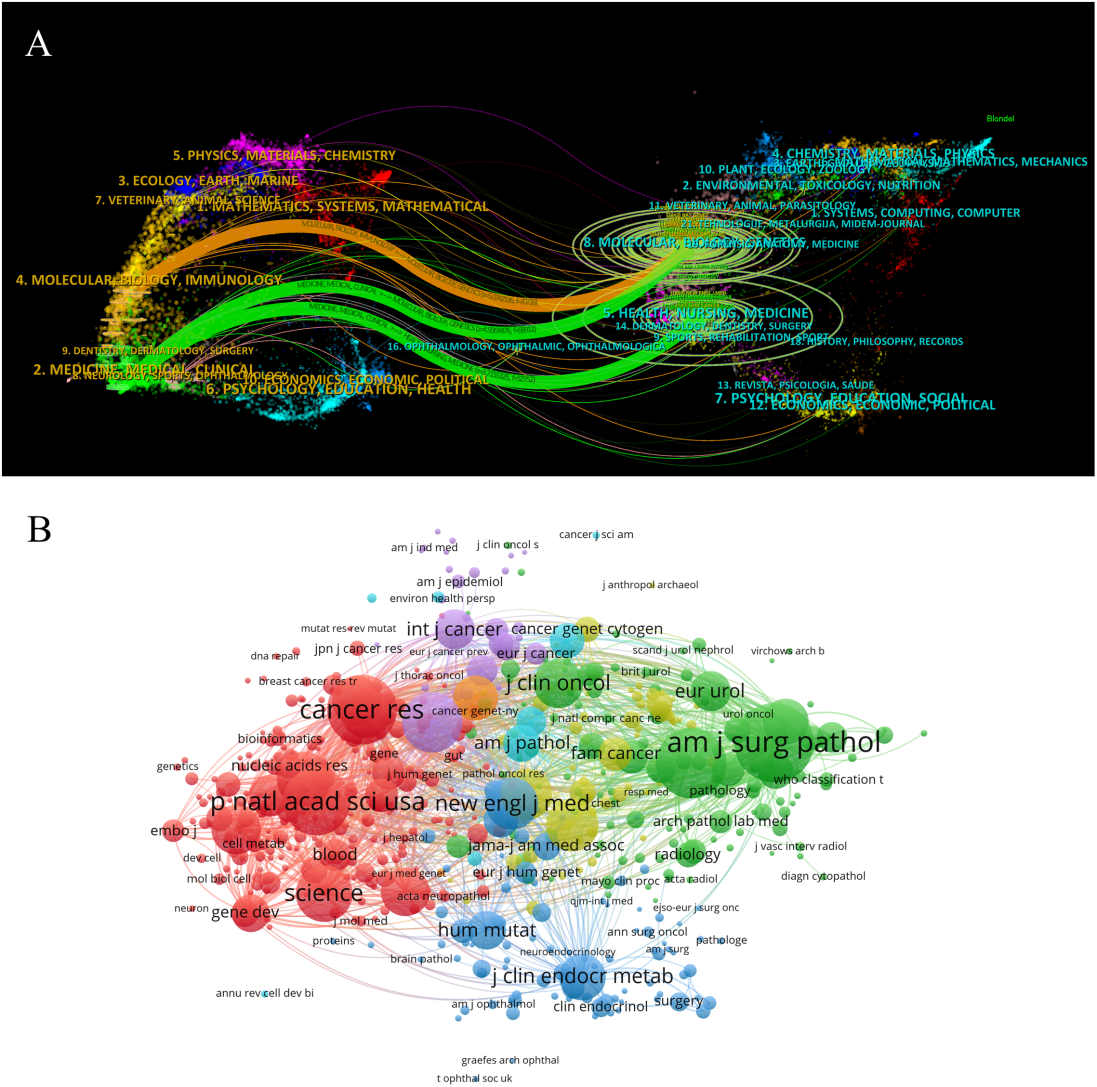
**(A)** CiteSpace-generated dual map overlap of journals that publish articles on hereditary renal carcinoma. **(B)** Visualization map overlaying the institutions that have a minimum of 20 articles.

Related articles have been published in 803 journals overall. The top ten journals for publication are displayed in **Table 4**. Their countries of origin are the United States, the Netherlands, and the United Kingdom. The journals that contributed the most were *Familial Cancer*, *Oncogene*, *Journal of Urology*, *American Journal of Surgical Pathology*, and *Journal of Medical Genetics*. Sixty percent of the top ten journals were in JCR Q1, and sixty percent of them had impact factors higher than 5.0. Among the top 10 journals, European Urology has the highest number of citations per article (228.38). A dual map illustrates the connection between citing and citing journals (**Figure 5A**). The dual map has three citation paths. The cited publications were categorized into 2 study areas: (1) molecular, biology, and immunology; and (2) medicine, medical, and clinical. Additionally, the cited studies were divided into two categories: (1) molecular, biology, and genetics; and (2) health, nursing, and medicine. Collaboration networks and clustering analysis of co-cited journals with more than 20 papers were illustrated (**Figure 5B**).

**Table 4:** Top 10 journals with most publications related to hereditary renal carcinoma.

Rank	Journal	Country	Publications	Total citations	Citation per paper	H-Index	IF (2022)	JCR (2022)
1	*Familial Cancer*	Netherlands	44	916	20.82	57	2.2	Q3
2	*Oncogene*	UK	39	3687	94.54	342	8	Q1
3	*Journal of Urology*	Netherlands	37	3212	86.81	256	6.6	Q1
4	*American Journal of Surgical Pathology*	USA	31	2536	81.81	210	5.6	Q1
5	*Journal of Medical Genetics*	UK	29	1757	60.59	170	4	Q2
6	*Genes Chromosomes & Cancer*	USA	25	811	32.44	119	3.7	Q2
7	*Human Molecular Genetics*	UK	24	2142	89.25	276	3.5	Q2
8	*Cancer Research*	USA	22	2215	100.68	449	11.2	Q1
9	*Clinical Cancer Research*	USA	22	2051	93.23	324	11.5	Q1
10	*European Urology*	Netherlands	21	4796	228.38	216	23.4	Q1

### 3.7 Analysis of top articles

To determine the top 100 papers, we ranked the articles based on their citation counts (**Supplementary Table S1**). 43,864 citations were made to the top 100 papers. Of the top papers, 330 (range: 183–2,417) were the median number of citations. The top ten articles with the most citations were all released in 2016 or before (**Table 5**). “A mitochondrial paradigm of metabolic and degenerative diseases, aging, and cancer: A dawn for evolutionary medicine,” published by Wallace et al. in 2005, possessed the most citations (2,417). “The 2016 WHO classification of tumours of the urinary system and male genital organs—Part A: renal, penile, and testicular tumours” published by Moch et al. in 2016, had the greatest average annual citation count (234.25) and the second-highest total number of citations (1,874).

**Table 5:** The 10 most cited papers in hereditary renal carcinoma from 2000 to 2023.

Rank	Title	Corresponding author	Journal	Year	Total citations	Average citations per Year
1	A mitochondrial paradigm of metabolic and degenerative diseases, aging, and cancer: A dawn for evolutionary medicine	Wallace, DC	ANNUAL REVIEW OF GENETICS	2005	2417	127.21
2	The 2016 WHO classification of tumours of the urinary system and male genital organs-part A: renal, penile, and testicular tumours	Moch, Holger	EUROPEAN UROLOGY	2016	1874	234.25
3	WNT signalling pathways as therapeutic targets in cancer	Moon, Randall T.	NATURE REVIEWS CANCER	2013	1486	135.09
4	Germline mutations in *FH* predispose to dominantly inherited uterine fibroids, skin leiomyomata and papillary renal cell cancer	Tomlinson, IPM	NATURE GENETICS	2002	1113	50.59
5	Iron and cancer: more ore to be mined	Torti, Suzy V.	NATURE REVIEWS CANCER	2013	1017	92.45
6	Von Hippel-Lindau disease	Lonser, RR	LANCET	2003	991	47.19
7	Hypoxia inducible factor-α binding and ubiquitylation by the von Hippel-Lindau tumor suppressor protein	Ratcliffe, PJ	JOURNAL OF BIOLOGICAL CHEMISTRY	2000	864	36
8	Comprehensive molecular characterization of papillary renal-cell carcinoma	Linehan, W. Marston	NEW ENGLAND JOURNAL OF MEDICINE	2016	834	104.25
9	Oxygen sensing, hypoxia-Inducible factors, and disease pathophysiology	Semenza, Gregg L.	ANNUAL REVIEW OF PATHOLOGY: MECHANISMS OF DISEASE	2014	787	78.7
10	The international society of urological pathology (ISUP) Vancouver classification of renal neoplasia	Srigley, John R.	AMERICAN JOURNAL OF SURGICAL PATHOLOGY	2013	767	69.73

### 3.8 Analysis of keywords

**Figure 6:**
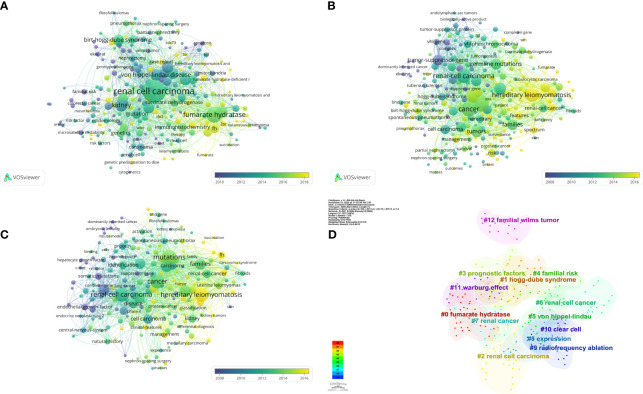
**(A)** Network visualization map of the cooperation connection of the keywords. **(B)** Network visualization map of the cooperation connection of the keywords appeared more than 15 times. **(C)** Network visualization map of the cooperation connection of the keywords from the 100 recently published papers. **(D)** Clusters of keywords: all nodes contained within a color block are members of the cluster, which is represented by a color block. The cluster scale decreases as the number of clusters increases.

**Figure 7:**
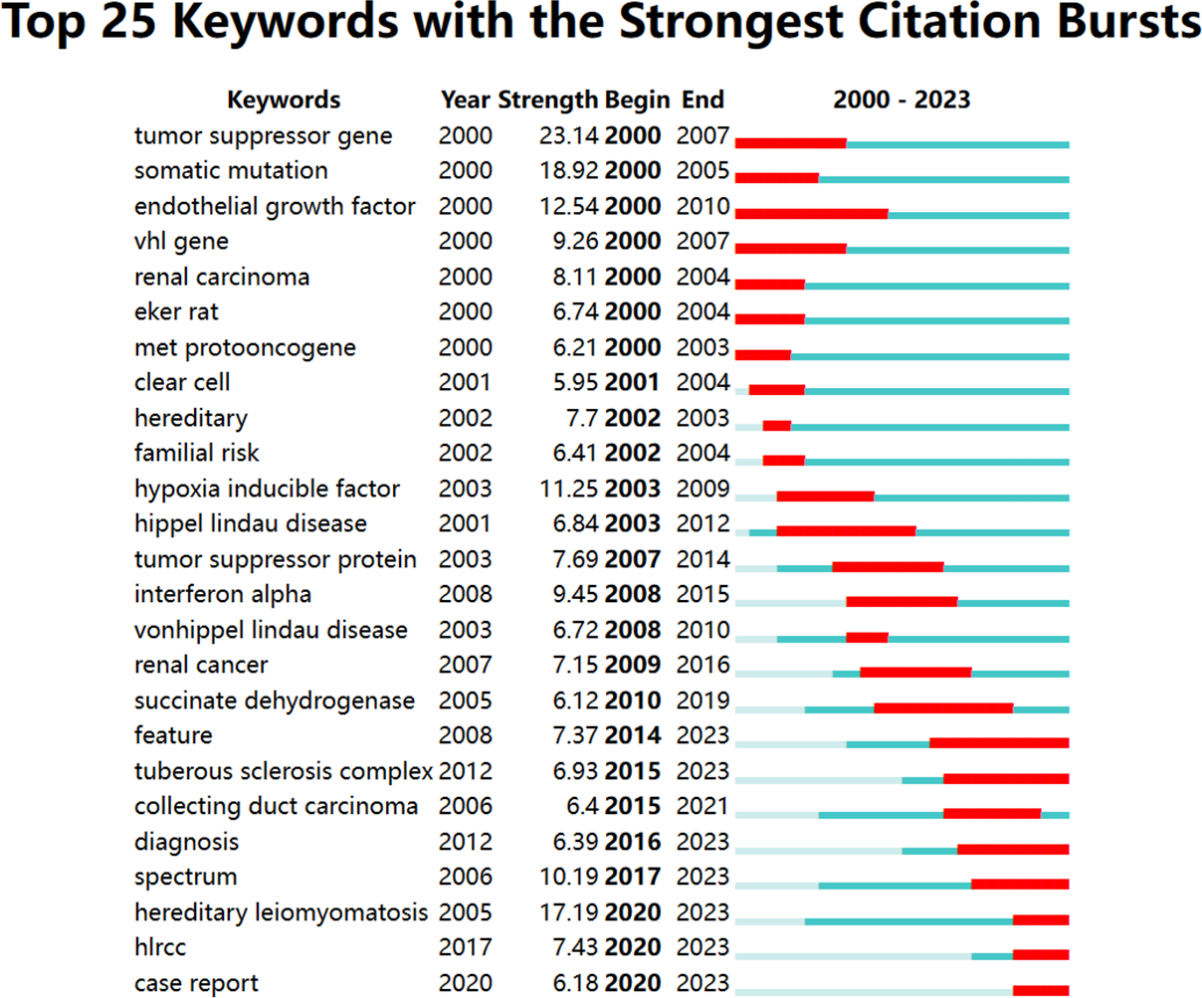
The top 25 keywords with the strongest citation bursts based on CiteSpace. The horizontal red stripes indicate the years in which the keyword was utilized most frequently. The horizontal green stripes indicate the years in which the keyword was utilized least frequently.

By looking at keyword co-occurrence across a vast number of literary works, we can categorize high-frequency keywords and assess the strength of links between keywords, revealing the study boundaries and internal structure of an academic subject. The top ten keywords, together with their frequencies, centrality, and first-occurrence timings, are shown in **Table 6**. The cooperation connection of the keywords was shown on a network visualization map (**Figure 6A**). The top search terms comprised “Renal cell carcinoma,” “mutation,” “cancer,” “hereditary leiomyomatosis,” “tumor,” “gene,” and “germline mutation.” The most recently occurring keywords in the top 10 were “hereditary leiomyomatosis” and “fumarate hydratase.” A cooperation and citation network analysis were performed on keywords that appeared more than 15 times (**Figure 6B**). The 100 recently published papers in prestigious journals were subjected to a keyword co-occurrence and citation network analysis (**Figure 6C**). We used cluster analysis to classify data by similarity based on keyword co-occurrence networks in order to get a deeper understanding of the fundamental knowledge structure of the field. As a result, we were able to generate the 10 keyword clusters (**Figure 6D**) in the domain of hereditary kidney cancer. The weighted mean silhouette S of the 10 clusters is 0.919, indicating both the excellent quality of the clustering results and the members’ strong homogeneity within the clusters. CiteSpace was used to determine the burst keywords based on the study of research hotspots. The burst map may show how keywords have gradually increased over time, which can be used to assess the period’s level of attention and research orientation. Research on the endothelial growth factor, tumor suppressor gene, and vhl gene between 2000 and 2010 was evident (**Figure 7**). However, after 2014, the tuberous sclerosis complex, diagnosis, and spectrum received enough attention to be considered potential future research frontiers and trends in the field of hereditary kidney cancer.

**Table 6:** High-frequency keywords.

Rank	Keyword	Frequency	Centrality	Year of first appearance
1	Renal cell carcinoma	503	0.19	2000
2	Mutation	386	0.1	2000
3	Cancer	310	0.1	2000
4	Hereditary leiomyomatosis	251	0.04	2005
5	Tumor	230	0.07	2001
6	Gene	217	0.07	2000
7	Germline mutation	198	0.04	2000
8	Family	190	0.05	2001
9	Fumarate hydratase	181	0.03	2005
10	Expression	168	0.09	2000

### 3.9 Analysis of research trends

**Figure 8:**
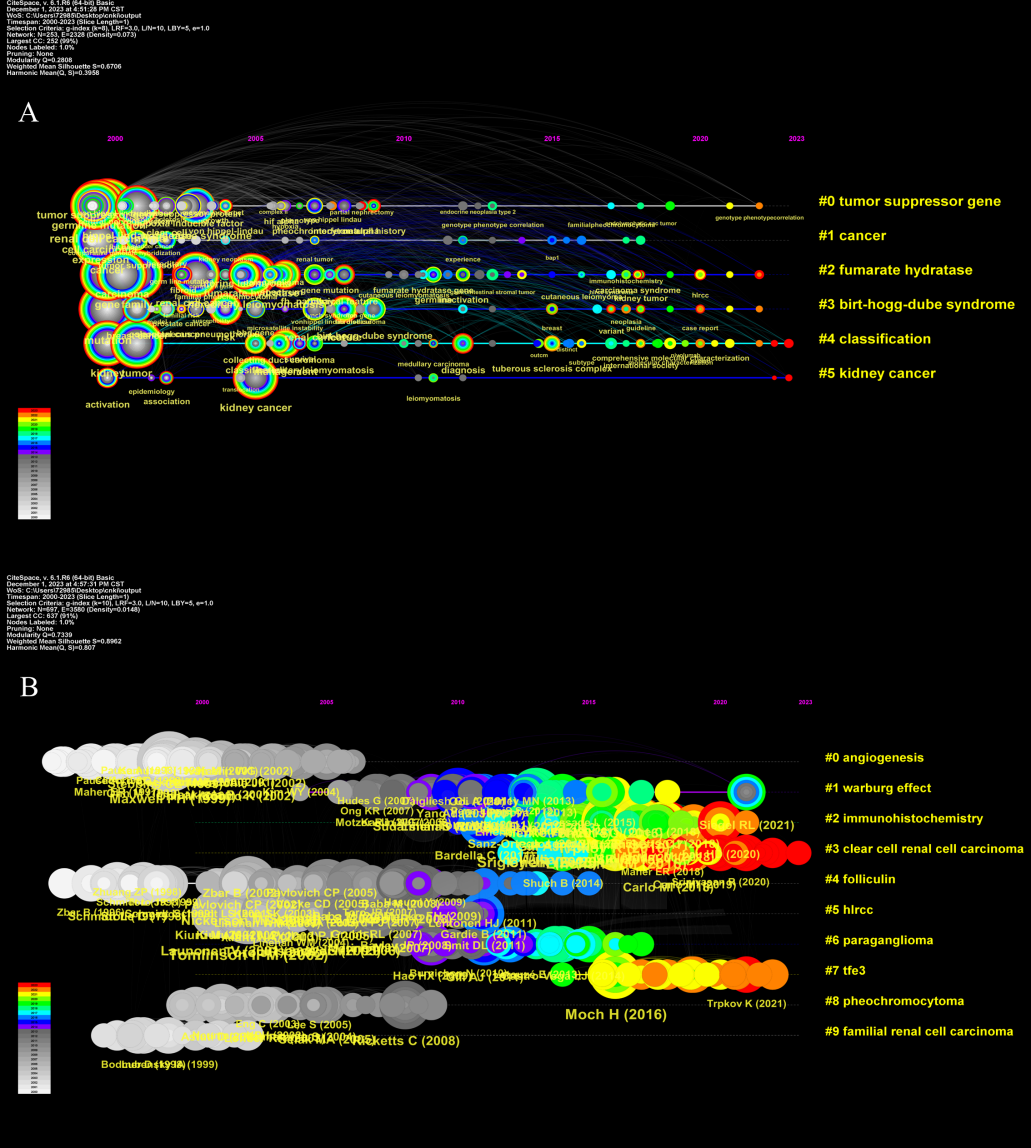
**(A)** The timeline view for co-cited keywords. The magnitude of the node corresponds to the citation count of the reference. Indicated by the curves connecting the nodes were co-citation relationships. **(B)** The timeline view for co-cited references. The node size represents the citation number of the reference.

**Figure 9:**
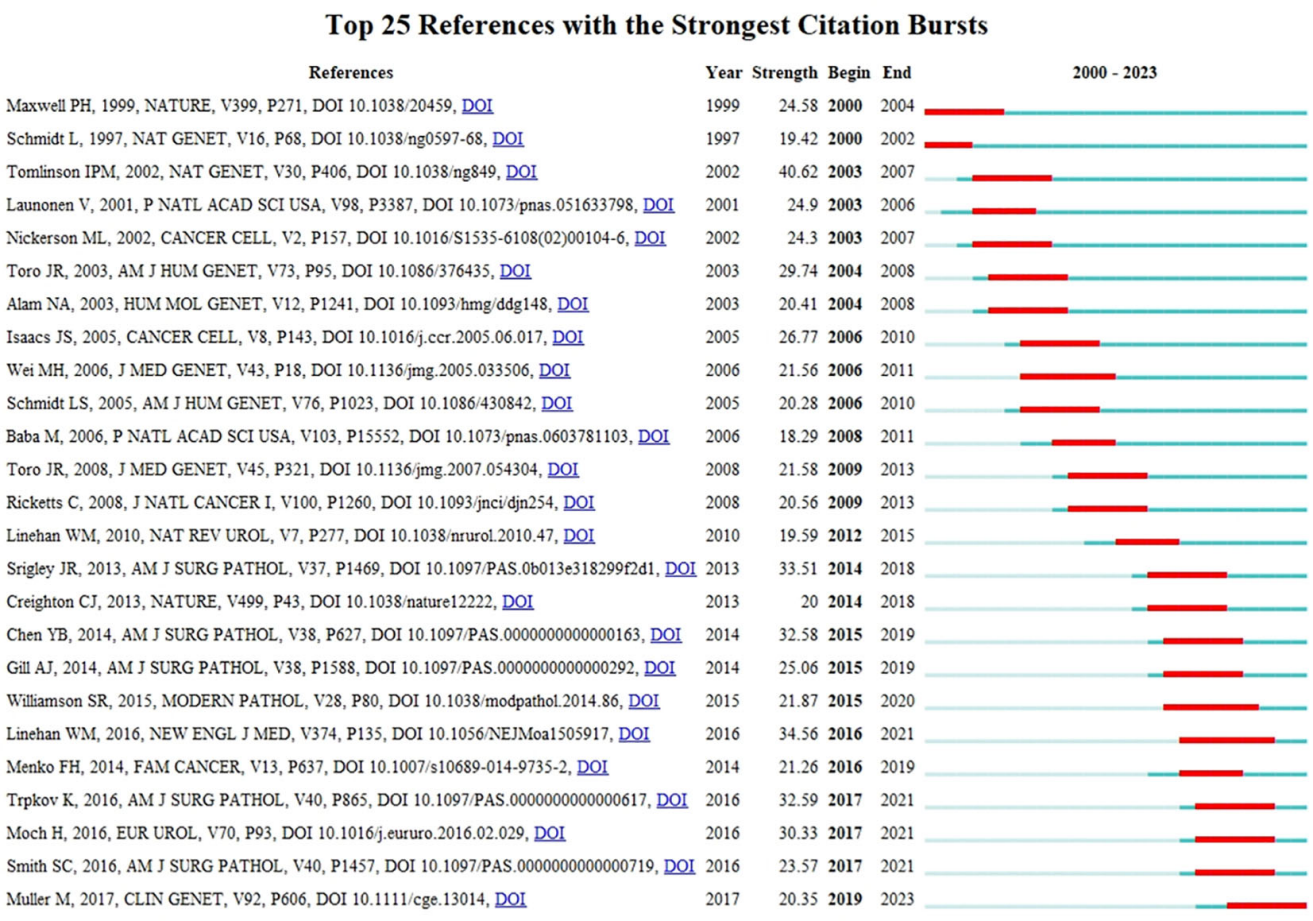
Visualization map of top 25 references with the strongest citation bursts generated by CiteSpace.

**Figure 8A** shows a historical perspective of the variance of co-cited keywords pertaining to hereditary kidney cancer. Five clusters were created from the keywords. **Figure 8B** shows a chronological perspective of the co-cited reference variation. Nine clusters were created from the references. Research hotspots were the themes with big yellow nodes, which stood for a lot of recent papers. **Figure 9** shows the burst map of the top 25 references. We employed VOSviewer to examine the co-cited references. This co-cited cooperation network between references cited more than 80 times is displayed (**Supplementary Figure S1**).

## 4 Discussion

A growing incidence of hereditary renal cancer has been identified due to the continuous advancement and refinement of diagnostic and gene detection technologies. The medical community has consistently been preoccupied with genetic renal cancer due to the inaccuracies in research concerning its etiology and therapeutic approaches. Although more research opportunities are allocated to hereditary kidney cancer each year, the annual literature volume in this field has not grown exponentially due primarily to the low incidence of the disease and the limited number of opportunities that have been devoted to it. Between the years 2000 and 2022, the quantity of relevant articles experienced a gradual yet steady increase, rising from 54 to 138. Due to its research institutions and scientists, the United States has consistently generated the greatest number of articles in medical research over the last two decades. Additionally, East Asian and European countries are significant due in part to their substantial investments in medical research and robust national economies. The United States houses six of the top ten article-publishing institutions, while developed European nations comprise the remaining four. As depicted in **Figure 3C**, these high-producing nations place considerable importance on international cooperation. The United States, England, Japan, Sweden, Finland, and various other nations were among the initial proponents of international cooperation, as illustrated in **Figure 3D**. Following this, France, Italy, and Spain exhibited greater levels of engagement, while East Asian and Eastern European countries have recently demonstrated an increasing inclination towards undertaking research pertaining to international cooperation. The significance of international collaboration in countries such as Brazil becomes evident when we focus on the top 100 quality-rated publications (**Figure 3E**). Literature originating from England exhibits a higher average number of citations per paper (**Table 1**), thereby providing a greater number of latecomers with valuable research references. The University of Birmingham, located in the United Kingdom, generated a total of 52 articles. At an average of 113.00 citations per article, it ranked as the second-most-cited institution, trailing only Harvard University (138.36 citations). Seven of the ten most prolific authors are from the United States, with the remaining three originating from the United Kingdom, France, and Japan. Lewis, an American author, has the highest H-index and is the most prolific. It is noteworthy that the top ten productive journals originate exclusively from the Netherlands, the United States, and the United Kingdom. The impact factor and number of citations are highest in *European Urology* from the Netherlands, whereas the H-index is highest in *Cancer Research*, which is based in the United States. The Dutch journal, *Familial Cancer*, concentrates on hereditary kidney cancer and has the most published literature among the top 10. However, both the number of citations for a single article and its impact factor are the lowest (**Table 4**). Prior to 2010, “tumor suppressor gene,” “somatic mutation,” “endothelial growth factor,” and “hypoxia inducible” were the strongest citation bursts over the last 23 years, as shown in **Figure 8**. Since 2017, “spectrum” and “hereditary leiomyomatosis” have become the strongest citation bursts of the last decade.

Significant progress has been made in the understanding of hereditary kidney cancer since the discovery of multiple germline syndromes that delineate the genetic and molecular characteristics of this disease (18). Consistent deficits in angiogenesis and metabolic signaling are observed in molecular defects associated with these inherited syndromes; these deficiencies are primarily caused by altered hypoxia signaling. VHL disease has been identified as the result of mutations in pVHL, the tumor suppressor gene product of the VHL gene. Recent findings from preclinical trials involving HIF-2α inhibitors and animal models indicate that ccRCC carcinogenesis is not exclusively attributed to HIF-2α, but rather involves collaboration with HIF-1α, potentially other pVHL targets, and additional mutated proteins. Ongoing research uncovers novel and intriguing functions of pVHL, the significance of which remains uncertain in both normal physiological and pathological contexts (19). As a result of research into the pathogenesis of VHL, advances in the treatment of diseases are also occurring. In August 2021, the FDA approved Belzutifan for the treatment of VHL-associated diseases, such as central nervous system hemangioblastomas (CNS HB) (20). Germline mutations in fumarate hydratase (FH), a fundamental enzyme in the Krebs cycle, is the main cause of HLRCC. When FH is lost, the Krebs cycle becomes less connected. This raises the level of fumarate and makes it harder for cells to oxygenate because NADH, an electron-transporting reducing substrate, is not present (21). Abnormally high levels of VEGF-A primarily control angiogenesis, which is the development of new blood vessels from already existing ones. This spike in VEGF-A is frequently triggered by tumor hypoxia during lesion expansion, but it can also arise from defects in the primary HIF pathway (22). Additionally, disruptions that occur during other stages of vascular development, i.e., PDGF-BB and Notch, contribute to and hasten the vascularization of tumors (23, 24). Moreover, Lynch syndrome (LS) has the potential to induce renal malignancies, including upper tract urothelial carcinoma (UTUC). In as few as 21% of newly diagnosed UTUC cases, LS may be the underlying cause that is not recognized. Carcinogenesis may result from errors that occur during the replication of repeated DNA sequences due to the inactivation of system genes in LS (25).

Recently, numerous bibliometric analyses have compiled the current research status of immunotherapy and targeted therapy for kidney cancer, along with outlining the latest advancements and future directions in kidney cancer research (26, 27). Research on the combination of targeted therapy and immunotherapy is currently a trending subject. Research in immune checkpoint inhibitors (ICIs), tumor vaccines, drug resistance, and predictive markers of therapy has garnered significant interest (28). Additionally, the recurrence and metastasis of renal cancer are areas of intense research. In addition to the aforementioned treatment modalities that are currently in use, cancer stem cell (CSC) treatment research has become a focal point in recent years. It may be critical to develop agents that target CSC-signaling pathways in order to benefit patients with renal cancer (29). Radiogenomics-mined preoperative imaging features are an excellent tool for optimizing surgical plans and informing us about disease characteristics. Furthermore, these features will serve as the subject of future clinical research on renal cancer (30).

Bibliometric analysis is a comprehensive, scientific, and objective technique utilized to track the development of research in a particular field and to serve as a comprehensive resource for subsequent scholars (31). This study provides a comprehensive understanding of the current state of research pertaining to hereditary kidney cancer over the last two decades. Latecomers in this field can use this article to comprehend the current state of research in this area and determine the most suitable course of action prior to initiating new investigations. This article also has several limitations. It was unable to include all publications prior to the publication of this article. Specific genetic illnesses have not been thoroughly researched in this research.

## 5 Conclusion

To summarize, this is the first bibliometric analysis of the literature on hereditary kidney cancer. We examined data on countries/regions, institutions, authors, journals, cited papers, keywords, and so on. We discovered that the number of articles in this category grew with time steadily. American authors and institutions have made the most contributions to this field. At the same time, the latest research hotspots have been unearthed. We hope that this study will be useful to other researchers working in the same subject. We will continue to investigate the pathophysiology of hereditary renal cancer in the future to provide evidence for the therapy of hereditary renal cancer.

## Data availability statement

The original contributions presented in the study are included in the article/**Supplementary Material**. Further inquiries can be directed to the corresponding authors.

## Author contributions

XL: Writing – review & editing, Writing – original draft, Software, Methodology, Investigation, Formal Analysis, Data curation, Conceptualization. MF: Writing – review & editing, Writing – original draft, Visualization, Supervision, Resources, Data curation. JL: Writing – review & editing, Writing – original draft, Software, Methodology, Investigation, Data curation. LZ: Methodology, Investigation, Formal Analysis, Data curation, Conceptualization, Writing – original draft, Validation, Software. PH: Writing – original draft, Visualization, Validation, Software, Methodology, Formal Analysis, Data curation, Conceptualization. YW: Writing – original draft, Visualization, Project administration, Methodology, Formal Analysis, Data curation. YZ: Writing – review & editing, Supervision, Investigation, Formal Analysis, Data curation, Conceptualization. SW: Writing – review & editing, Visualization, Validation, Supervision, Resources, Project administration, Formal Analysis. CzL: Writing – review & editing, Visualization, Validation, Supervision, Resources, Project administration. ClL: Writing – review & editing, Validation, Supervision, Methodology, Funding acquisition, Data curation.

## Supplementary material

Supplementary Figure 1 Co-cited reference collaboration network visualized by VOSviewer.
