# Supplementary figures and images for: A 23-year bibliometric analysis of the development of global research on hereditary renal carcinoma
Source: Front Oncol. 2024 Jun 3;14:1364997. doi: 10.3389/fonc.2024.1364997 (PMC11180816; doi:10.3389/fonc.2024.1364997)

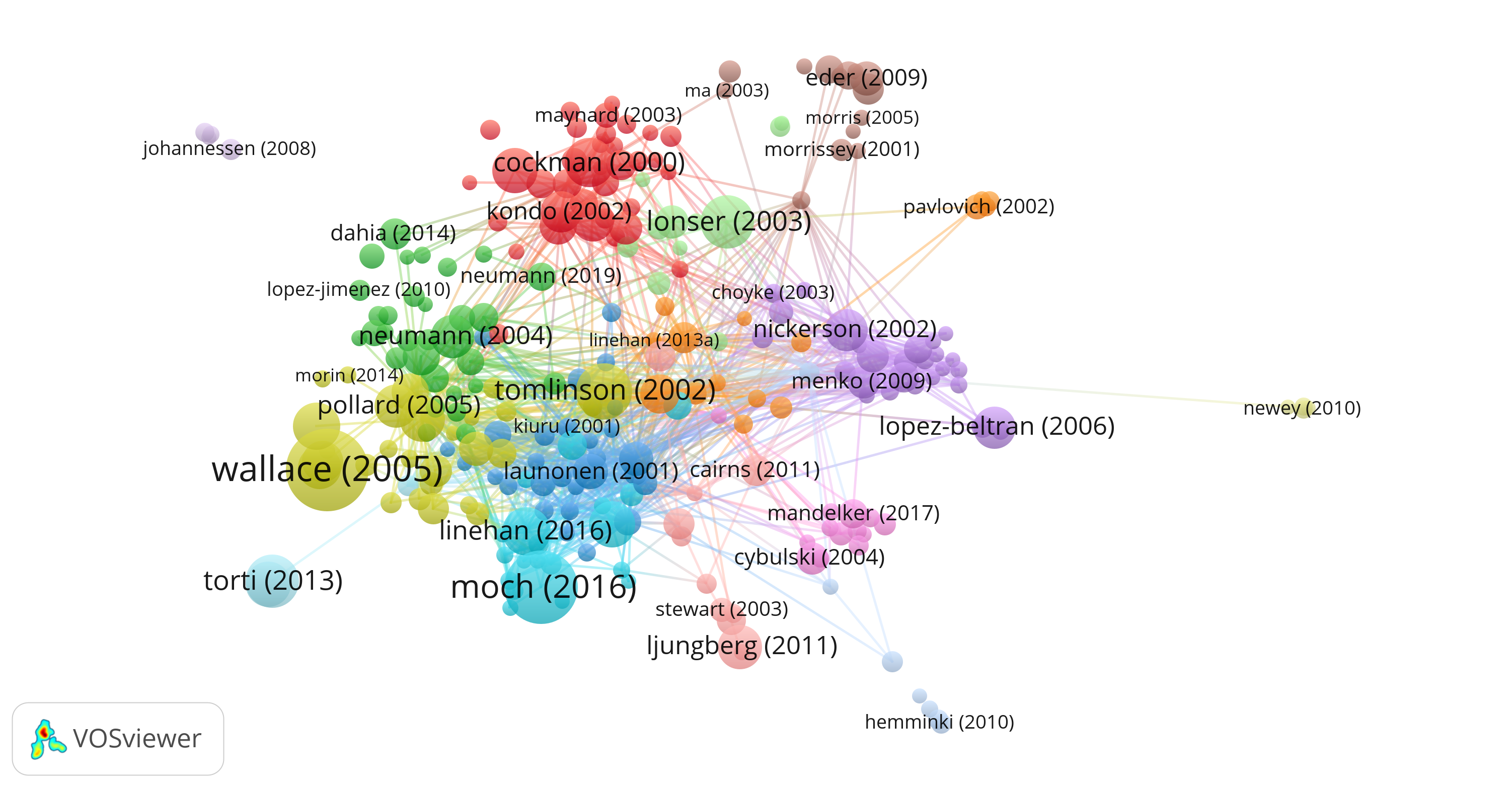

Supplement: Supplementary Figure 1 — Co-cited reference collaboration network visualized by VOSviewer. [file Image_1.png]
